# Analytical Study of the Propagation of Fast Longitudinal Modes along wz-BN/AlN Thin Acoustic Waveguides

**DOI:** 10.3390/s150202525

**Published:** 2015-01-23

**Authors:** Cinzia Caliendo

**Affiliations:** Istituto di Acustica e Sensori Corbino, IDASC-CNR, Via del Fosso del Cavaliere 100, 00133 Roma, Italy; E-Mail: cinzia.caliendo@idasc.cnr.it; Tel.: +39-6-4993-4741; Fax: +39-6-4548-8061

**Keywords:** wz-BN, AlN, GHz range frequency, liquids, high coupling efficiency

## Abstract

The propagation of the fundamental symmetric Lamb mode S_0_ along wz-BN/AlN thin composite plates suitable for telecommunication and sensing applications is studied. The investigation of the acoustic field profile across the plate thickness revealed the presence of modes having longitudinal polarization, the Anisimkin Jr. plate modes (AMs), travelling at a phase velocity close to that of the wz-BN longitudinal bulk acoustic wave propagating in the same direction. The study of the S_0_ mode phase velocity and coupling coefficient (K^2^) dispersion curves, for different electrical boundary conditions, has shown that eight different coupling configurations are allowable that exhibit a K^2^ as high as about 4% and very high phase velocity (up to about 16,700 m/s). The effect of the thickness and material type of the metal floating electrode on the K^2^ dispersion curves has also been investigated, specifically addressing the design of an enhanced coupling device. The gravimetric sensitivity of the BN/AlN-based acoustic waveguides was then calculated for both the AMs and elliptically polarized S_0_ modes; the AM-based sensor velocity and attenuation shifts due to the viscosity of a surrounding liquid was theoretically predicted. The performed investigation suggests that wz-BN/AlN is a very promising substrate material suitable for developing GHz band devices with enhanced electroacoustic coupling efficiency and suitable for application in telecommunications and sensing fields.

## 1. Introduction

Electroacoustic devices based on the propagation of surface and bulk acoustic waves (SAW and BAW) are known for their wide range of applications in chemical, biological and physical sensing fields. When these devices are required to operate in a liquid environment, they must involve the propagation of specific types of acoustic waves that do not radiate energy into the liquid. Love waves, surface transverse waves (STW), shear horizontal acoustic plate modes (SHAPM) and shear horizontal SAW (SHSAW) are examples of acoustic waves whose shear horizontal polarization ensures no coupling between the liquid and the elastic propagating medium. Waves with elliptical polarization, such as the Rayleigh-like waves, are impractical for use in liquid environments as a consequence of the high energy loss in the liquid, both by radiating compressional waves into the fluid and due to frictional losses in the fluid. The fundamental antisymmetric Lamb mode, A_0_, while being elliptically polarized, can travel along thin plates that are in contact with a liquid for a limited plate thickness range corresponding to mode velocities lower than the compressional velocity v_liq_ of the surrounding liquid medium. The fundamental symmetric Lamb mode, S_0_, as well as the higher order symmetrical and antisymmetrical modes are elliptically polarized and have a velocity higher than that of the surrounding liquid medium, and thus, they are not suitable for sensing applications in liquids, except for some special cases. These cases include the linearly-polarized modes, such as the longitudinally-polarized Anisimkin Jr. modes (AMs), with a dominant longitudinal displacement component U_1_ with a constant amplitude along the whole depth of the plate, the shear components, U_2_ and U_3_, being at least 10-times less than U_1_ at any plate depth. AMs were first discovered in quartz plates [1] and then in LiNbO_3_, Te [2], LiTaO_3_, Ba_2_NaNb_5_O_15_, KH_2_PO_4_, Li_2_B_4_O_7_, TeO_2_, PbMoO_4_, KH_2_PO_4_ and ZnO [3,4] and, recently, in GaPO_4_ [5]. The AMs, while having a phase velocity close to that of the longitudinal bulk acoustic wave (LBAW) traveling in the same direction, can be suitable for the development of high-frequency electroacoustic devices if implemented on fast materials, such as BN and AlN. Boron nitride (BN) is a group III–V compound semiconductor, and wurtzite (wz-BN) and cubic BN (zb-BN) are two representative crystal structures. The former is chemically inert and thermally stable and has been widely used as an electrical insulator and heat-resistant material. The latter, which is a super-hard material with properties similar to diamond, including chemical inertness, shows excellent properties, such as a high melting temperature, high thermal conductivity and chemical stability. zb- and wz-BN films are currently grown by sputtering (DC or RF) and by hollow cathode arc evaporation apparatus on Si(001) and Si(111), respectively [6–9]. AlN is the fastest piezoelectric material among those that can be grown in thin film form: compared to ZnO, for example, AlN films show higher SAW velocity (5607 m/s, as opposed to 2682 m/s), higher hardness (17.7 GPa, as opposed to 4.7 GPa) and thermal conductivity (2.8 W·cm^−1^·°C^−1^, as opposed to 0.6 W·cm^−1^·°C^−1^) and a slightly lower electroacoustic coupling coefficient K^2^ (0.3%, as opposed to 0.97%). The AlN resistance to high temperature and to caustic chemicals make it the ideal candidate for the development of electroacoustic devices able to survive in harsh environment. The present paper investigates the acoustic wave propagation along wz-BN/AlN thin composite plates that are suitable for the development of high-frequency electroacoustic devices for telecommunications and sensing applications [10].

## 2. SAW and BAW Propagation along BN and AlN

The SAW phase velocity in zb-BN is highly affected by the material anisotropy, as well as the BAW; on the contrary, wz-BN and c-AlN are isotropic in the c-plane, and hence, both the SAW and BAW velocities are unaffected by the propagation direction in this plane. The SAW propagating along the AlN and wz-BN c-plane is a Rayleigh-type wave that has only two non-null rapidly-damped displacement components, U_1_ and U_3_, lying in the sagittal plane. The K^2^ of the Rayleigh-like waves, as well as the velocities of the SAW and BAW propagating along the <100> direction of AlN and BN crystals are listed in Table 1. The acoustic wave velocity calculations were performed by using MATLAB software in the lossless approximation; the BN and AlN material data were deduced by [11,12].

As can be seen from Table 1, wz-BN is a weakly piezoelectric material showing very high SAW and BAW velocities, which make this material attractive for the development of high-frequency electro-acoustic devices.

## 3. Acoustic Wave Propagation along BN and BN/AlN Plates

### 3.1. BN Plates

**Figure 1.**
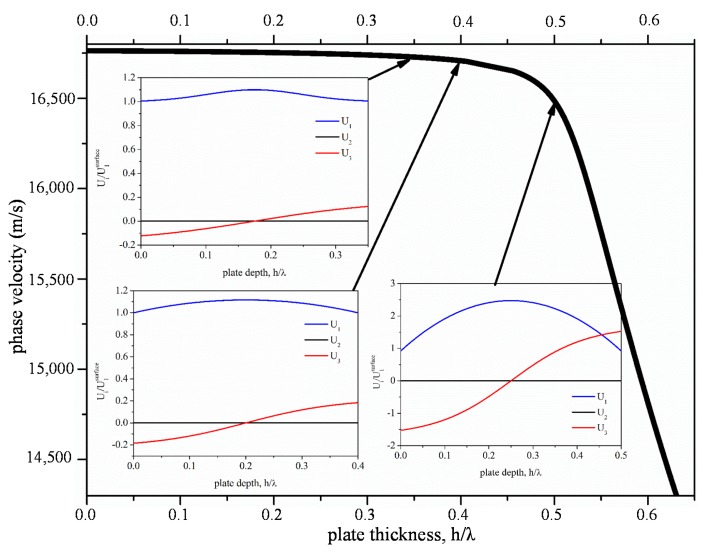
Phase velocity of the S_0_ mode *vs.* h_BN_/λ; the insets show the acoustic field profile for h_BN_/λ = 0.35, 0.4 and 0.5.

Figure 1 shows the phase velocity of the fundamental symmetric Lamb mode, S_0_, *vs.* the bare wz-BN plate thickness normalized to the wavelength, h_BN_/λ. The dispersion curve is characterized by a low dispersion region, where the velocity is almost constant and very close to v_LBAW_, followed by a drop in velocity. In the flat dispersion region, the S_0_ mode transforms first to the AM counterpart with constant and dominant longitudinal displacement component U_1_, while U_2_ = 0 and U_3_ ≤ 10%·U_1_. With increasing the plate thickness, the mode transforms to the quasi-longitudinal (QL) counterpart: the longitudinal displacement component is no longer constant through the thickness of the plate, but it is still dominant over the other two components (U_2_ = 0 and U_3_ ≪ U_1_). For higher plate thicknesses, when the S_0_ mode velocity approaches the shear horizontal BAW velocity, the shear horizontal component U_2_ largely dominates U_1_ and U_3,_ but this wave is electrically uncoupled. The insets of Figure 1 show the three particle displacement components, U_1_, U_2_ and U_3_, *vs.* the BN plate depth, for three different plate thicknesses, h_BN_/λ = 0.35, 0.4 and 0.5. At h_BN_/λ = 0.35, the QL mode (QLM) propagates, while with increasing h_BN_/λ, the U_3_ contribution slowly increases and becomes comparable to that of U_1_, while the displacement component U_2_ remains equal to zero. For h_BN_/λ > 1, the mode begins to act as a Rayleigh wave propagating on the surfaces of the plate.

**Figure 2.**
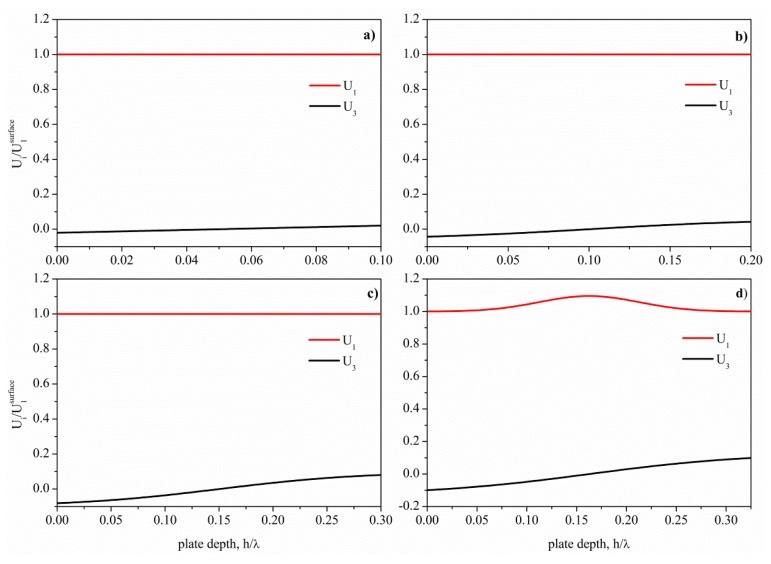
Depth profile of the AMs propagating along BN plates of different thicknesses normalized to the wavelength: (**a**) h_BN_/λ = 0.1; (**b**) h_BN_/λ = 0.2; (**c**) h_BN_/λ = 0.3; and (**d**) h_BN_/λ = 0.325.

The theoretical investigation of the mode field profile across the depth of the wz-BN thin bare plate revealed that the AMs propagate in the limited h_BN_/λ range up to 0.325, with velocities from 16,760 (h_BN_/λ = 0.1) to 16,735 m/s (h_BN_/λ = 0.325). Figure 2 shows the depth profile of the AMs for four BN plate thicknesses: the displacement components U_1_ and U_3_ are normalized to the U_1_ value at the surface. The shear horizontal component U_2_ is not shown in Figure 2, as it is null. For h_BN_/λ > 0.325, U_3_ increases, and U_1_ is no longer constant through the plate depth; the acoustic field profile of the S_0_ mode changes according to the behavior shown in the insets of Figure 1.

Unfortunately, the piezoelectricity of BN is weak, thus BN-based electroacoustic devices are impractical without the addition of a thin piezoelectric film, such as AlN, whose technology is well established nowadays.

### 3.2. BN/AlN Composite Plates

The BN/AlN acoustic waveguide that we want to investigate consists of an acoustically thin suspended membrane that can be fabricated by a conventional bulk micromachining technique of a specifically orientated Si wafer. In addition to the Si substrate, the wz-BN thin film is to be used as an etch stop and to form the underlying membrane of the device. A piezoelectric AlN film is deposited on the BN layer, followed by a metallic layer (typically Al, Mo or Pt) on which the interdigital transducers (IDTs) are photolithographically patterned to generate and detect the propagating acoustic waves. The acoustic waves are generated by the application of an alternating voltage signal to the IDTs patterned on the piezoelectric substrate; the IDTs geometry dictates the wavelength of the excited wave, (λ = IDTs' period length), and the phase velocity of the wave dictates the device frequency, f = v/λ. The fabrication procedure of the BN/AlN-based acoustic waveguide is compatible with the semiconductor processing techniques, thus offering the advantage of providing the monolithic integration of the device with the signal processing electronics.

**Figure 3.**
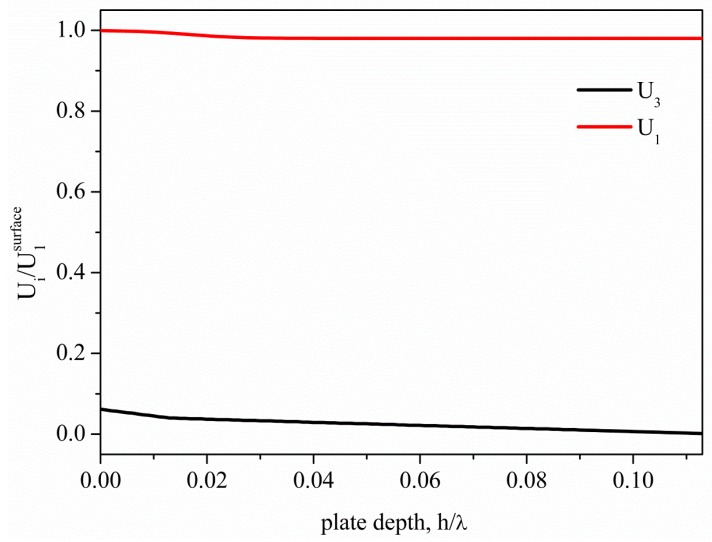
Depth profile of the AM propagating along the BN/AlN composite plate with h_BN_/λ = 0.1 and h_AlN_/λ = 0.013.

AMs propagate along BN/AlN composite plates only for very small AlN thicknesses that depend on the BN underlayer thickness. As an example, Figure 3 shows the AM field profile in the BN/AlN plate for h_BN_/λ = 0.1 and h_AlN_/λ = 0.013, 0.013 being the maximum AlN thickness that still allows the propagation of the AM through the composite plate with h_BN_/λ = 0.1. As can be seen, the presence of the AlN layer weakly affects the AM depth profile, while for h_AlN_/λ > 0.013, the AM converts into a QLM. For h_AlN_/λ < 0.013, the AM field profile along the plate depth is unaffected by the presence of the AlN layer, being U_1_ = 1 across the plate depth. For h_BN_/λ = 0.2, 0.3 and 0.325, the maximum h_AlN_/λ values compatible with the AMs propagation along the BN/AlN composite plates are 0.01, 0.004 and 0.001, respectively.

**Figure 4.**
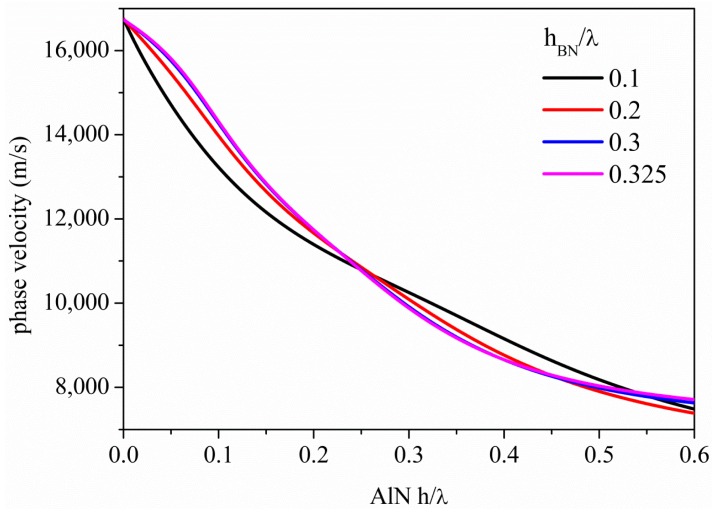
Phase velocity *vs.* AlN h/λ of the S_0_ mode along the BN/AlN composite plates, h_BN_/λ being the running parameter.

Figure 4 shows the S_0_ mode dispersion curves, *i.e.*, the phase velocity *vs.* h_AlN_/λ, along the BN/AlN composite plates, for different BN plate thicknesses (h_BN_/λ = 0.1, 0.2, 0.3 and 0.325). As can be seen, for h_AlN_/λ∼0, the velocity equals that of the bare BN plate, but increasing the h_AlN_/λ, the mode phase velocity gradually decreases and asymptotically reaches the AlN SAW velocity for h_AlN_/λ > 1. The presence of the AlN film lowers the mode velocity, but has the great advantage of increasing the coupling coefficient of the composite plate with respect to that of the bare BN plate, as shown in the next paragraph.

### 3.3. The Coupling Coefficient Dispersion Curves

**Figure 5.**
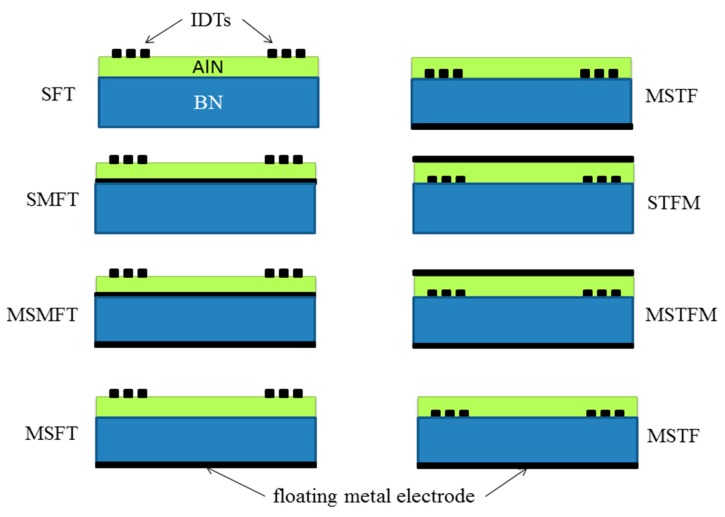
Cross-sections of the eight coupling configurations: the green, blue and black layers refer to the AlN (F, film), BN (S, substrate) and metal films (M, metal); the IDTs (T, transducers) are also shown.

The bare wz-BN plate is impractical for the development of an electroacoustic device, as its K^2^ is weak, but this can be improved by covering the plate with a thin piezoelectric film, such as AlN, and selecting the proper electrical boundary conditions. The waveguide modes are excited and detected by the IDTs patterned on one surface of the AlN film: the application of an alternating electrical potential to one of the IDTs imposes an alternating strain field in the AlN film, which launches a mechanical wave into the composite plate. When the acoustic mode reaches the second transducer, it generates an electrical signal, which conveys information about the attenuation and phase delay experienced by the wave as it travels through the plate. By placing the IDTs on the AlN surface or at the BN/AlN interface, with or without one or two metallic layers opposite the IDTs, eight different electroacoustic coupling structures can be implemented, as depicted in Figure 5.

**Figure 6.**
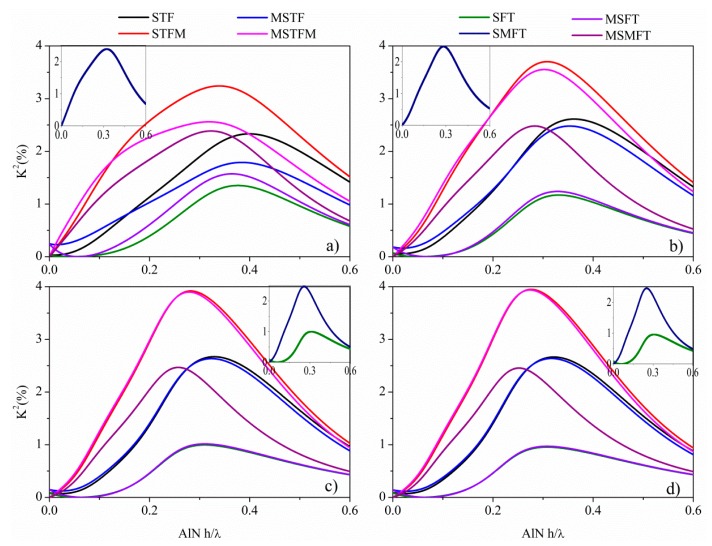
K^2^ *vs.* h_AlN_/λ dispersion curves of the eight BN/AlN-based coupling structures: (**a**), (**b**), (**c**) and (**d**) refer to h_BN_/λ = 0.1, 0.2, 0.3 and 0.325, respectively.

The configuration called substrate/film/transducer (SFT) refers to a composite plate with the IDTs positioned on the AlN free surface: when a floating metallic plane (M, metal) is placed at the AlN/BN interface and/or at the BN free surface, the configurations are called SMFT, MSMFT or MSFT. The configuration called substrate/transducer/film (STF) refers to a composite plate with the IDTs positioned at the AlN/BN interface: when the metallic plane is positioned at the AlN free surface and/or at the BN free surface, the configurations are called STFM, MSTFM or MSTF. The K^2^ is a measure of the electrical to acoustic energy conversion efficiency, and it was approximated to be 2[(v_f_ − v_m_)/v_f_], where v_m_ and v_f_ are the velocities calculated in the short-circuited and free boundary condition, in the approximation of an infinitesimally-thin perfectly-conductive floating electrode and IDTs. The K^2^ dispersion curves were studied for the eight BN/AlN-based configurations, for different BN and AlN thicknesses normalized to the acoustic wavelength, h_BN_/λ and h_AlN_/λ, in the range 0.00001 to 0.6 with a step size of 0.0001. These curves were obtained taking into account only the electric loading from the IDTs and the metal floating plane. Figure 6 shows the K^2^ *vs.* h_AlN_/λ dispersion curves of the eight coupling structures, for h_BN_/λ = 0.1, 0.2, 0.3 and 0.325: the insets show those K^2^ dispersion curves that are not clearly distinguishable as a consequence of their partial overlapping with other curves. As can be seen, the K^2^ of the BN/AlN plate depends very strongly on the electrical boundary conditions and on the BN and AlN layers thickness, and it reaches values higher than that of the single AlN piezoelectric film (0.28%).

For h_AlN_/λ∼0, the K^2^ of each configuration is small, but with increasing the AlN thickness, it increases and reaches a peak; for h_AlN_/λ ≫ 1, the K^2^ moves toward the K^2^ of the SAW that propagates in the (0001) plane of the AlN semi-infinite substrate. A K^2^ as high as ∼4% can be obtained at h_AlN_/λ ∼ 0.27 with the STFM and MSTFM structures on h_BN_/λ = 0.325, for v∼10,300 m/s. In general, the coupling configurations with the metal floating plane placed on one surface of the AlN layer (STFM, MSTFM) have a higher electromechanical coupling coefficient than that of the configurations with a free AlN surface (SFT, MSTF, MSFT), because the electric field strength through the AlN thin plate is higher than that between the IDT electrodes. The metallization on the AlN side opposite the IDTs strongly enhances the vertical electric field in the AlN layer.

The STFM and MSTFM coupling structures are the most efficient between the eight, and Table 2 lists the highest K^2^ values that are obtainable by these two configurations for different BN/AlN thicknesses, together with the corresponding phase velocity.

All of the data listed in Table 2 refer to a Lamb-like wave with two non-null particle displacement components, U_1_ and U_3_; thus, such configurations are not suitable for operation in liquids, but only in a vacuum or air.

As mentioned previously, the AMs can propagate along the BN/AlN composite plate only for a limited AlN thickness range that depends on the underlying BN layer thickness. Table 3 lists the highest AlN thicknesses that still allow the AMs' propagation on the BN/AlN plates, the K^2^ and the velocity of the most efficient coupling configuration between the eight.

The AMs K^2^ is lower than that of the Lamb modes of Table 2, but the velocity is greater and suitable for achieving very high operation frequencies (in the GHz band) with micron feature-sized IDTs.

All of the calculations listed in Tables 2 and 3 were performed taking into account only the electrical loading of the IDTs and the metal plane. A subsequent investigation accounting both for the electrical and mass loading of the IDTs and the metal electrode was performed; the effect of the metal electrode thickness and material type (Pt, Mo and W) was studied for the S_0_ and AMs-based coupling structures listed in Tables 2 and 3. Different metal thickness ranges were studied relative to the AMs (h_m_/λ = 0.0001 to 0.005, step size of 0.0001) and S_0_ mode (h_m_/λ = 0.001 to 0.01, step size of 0.001): the ranges were properly chosen, so as not to excite the propagation of unwanted acoustic modes. In the case of the AMs, the K^2^ does not change with respect to the value calculated taking into account only the electric loading effect, but it decreases when h_m_/λ is comparable with h_AlN_/λ, whatever the metal type. In the case of the S_0_ modes, a quite small improvement (2.65%) on K^2^ can be obtained for the STFM configuration for the Mo electrode with thickness h_m_/λ = 0.007 and for h_BN_/λ = 0.2 to 0.325; the highest improvement in K^2^ (from 7% to 10%) can be obtained for the MSTFM configurations with the W electrode, for h_m_/λ = 0.09.

## 4. BN/AlN-Based Acoustic Waveguides for Sensing Applications

When an electroacoustic device is used for sensor applications, its output signal (a frequency and/or attenuation shift) is produced by the interaction between the acoustic wave (AW) and the chemical, biological or physical input to be measured. This interaction perturbs the AW characteristics, such as velocity and/or attenuation, through mechanical, acoustoelectric or viscoelastic coupling between the input stimulus to be measured and the acoustic wave field. For example, when an AW device is used for chemical vapor sensing, the wave propagation path is covered by a gas-specific sensing film: when this film is exposed to a gas, mechanical and electrical perturbations in the film will produce a shift in the resonant frequency of the acoustic wave device oscillator. Alternatively, by measuring the AW attenuation and the frequency shifts, one can determine the viscosity and density of a fluid that contacts these sensors. Generally the sensors based on electroacoustic devices are small, relatively inexpensive, quite sensitive and able to measure a wide variety of different input quantities [13].

### 4.1. Mass Sensors

The most common sensing application of the electroacoustic devices is based on the gravimetric principle for mass detection. A mass accumulation on the device surface changes the surface density of the propagating medium, hence resulting in a wave velocity shift. A mass sensor based on a high-frequency device exhibits superior performances: the higher the frequency, the higher the sensor sensitivity and resolution. If the added mass consists of an ideal thin elastic film that moves synchronously with the oscillating surface, the fractional velocity change-to-added mass ratio defines the sensor's gravimetric sensitivity S = [(v − v_l_)/v_l_]/m, m = ρ·h, ρ and h being the added layer's mass density and thickness, v and v_l_ the unloaded and mass-loaded plate's phase velocity. Tables 2 and 3 list the sensitivity data of the S_0_ Lamb modes and the AMs propagating along the BN/AlN structures. Since the gravimetric detection can occur either on the AlN or BN plate surface, two normalized gravimetric sensitivity values, S and S′, were calculated for each AM and S_0_-based configuration. S has been calculated as S = [(v − v_0_)/v_0_]/(ρh/λ), where v_0_ and v are the wave velocities along the bare plate and the plate covered by a thin film, ρ is the film mass density and h/λ is the film thickness normalized to the wavelength. As can be seen, the AMs are more sensitive than the S_0_ modes, and the AlN side of the composite plate is more sensitive than the BN side, irrespective of the thickness of both materials, the energy confinement at the BN side being lower than that at the AlN side. As an example, for λ = 10 μm, the AMs-based mass sensor (BN = 0.1λ = 1 μm, AlN = 0.013λ = 0.13 μm) shows S = −123 m^2^/Kg, K^2^ = 0.25% and f = 1613 MHz, and the S_0_-based sensor (BN = 0.325λ = 3.25 μm, AlN = 0.274λ = 2.74 μm) shows S = −58 m^2^/Kg, K^2^ = 4% and f = 1028 MHz. These sensors show superior performances as compared to the SAW mass sensors implemented on STx-quartz, yx-, yz-and 128° tilted LiNbO_3_, for which S = −36, −29, −21 and −31 m^2^/Kg, K^2^ = 0.12, 1.56, 4.82 and 4.60% and f = 316, 377, 340 and 380 MHz, respectively [14].

### 4.2. Viscosity Sensor

The AMs are a very attractive choice for sensing applications in liquids, as they have only one source of attenuation, the friction between the surface plate and the adjacent liquid, while the S_0_ wave propagating in thick plates bordered by a liquid becomes a leaky wave due to the significant value of particle displacement normal to the plate surface and the radiation of compressional waves into the liquid. By monitoring the perturbations of the AMs propagation characteristics, such as the frequency and attenuation, one can measure the liquid density, viscosity, conductivity, permittivity and small mass changes. Although the AMs' gravimetric sensitivity was calculated in vacuum, it is a parameter of primary importance in the calculation of the sensitivity of a sensor for determining the viscosity of a fluid that contacts the device surface. The oscillation of the device surface contacting the liquid environment leads to entrainment of a thin liquid boundary layer near the interface that vibrates with a phase lag and an attenuated amplitude with respect to the surface vibration. The liquid layer velocity profile has an in-phase component that contributes to the viscous mass loading: a velocity shift arises from mass loading by the entrained viscous liquid layer of a thickness equal to the decay length of the AM field into the liquid, δ = [2η/ρω]^1/2^, ρ and η being the liquid density and shear viscosity, ω = 2πf and m′ = [ρ_l_η/2ω]^1/2^ is the liquid mass that vibrates in phase with the plate mode. The velocity out-of-phase component contributes to the mode attenuation arising from the power dissipation in the liquid. The viscous coupling of the AMs to an adjacent Newtonian liquid results in both a change in the propagation velocity Δv/v = (S/2ω)·[ηωρ_l_/2]^1/2^ and attenuation α = (S/2v_0_)·[ωρ_l_η/2]^1/2^ of the mode, and these changes are proportional to the mass sensitivity of plate mode velocity S [15,16]. As an example, for λ = 100 μm, at a frequency of 160 MHz and for 50% glycerin/water mixture (η ≅ 6.879 mPa·s, ρ ≅ 1.144 g/cm^3^), δ ≅ 0.1 μm, Δv/v_0_ = 0.08% and α = 0.34 dB/cm, for the BN(0.1λ)/AlN(0.013λ)-based MSTFM configuration. This specific AM-based configuration, in addition to the advantage of having the highest mass sensitivity among the others, allows one to make measurements of the liquid viscosity that are not affected by the electric conductivity of the liquid that is being characterized, due to the presence of the metal electrode on the sensing side that short-circuits the electric field outside the plate. As the ability to detect perturbations on the plate side is affected by the materials' combination and the electrical boundary conditions, the measurements of the electrical conductivity of the liquid can be carried out using those configurations that provide for the direct contact between the liquid and the unmetallized surface of the BN/AlN-based configuration.

## 5. Conclusions

We report on the observation of very high velocity guided modes along thin BN/AlN composite plates, which exist when the plate thickness is about an order of magnitude smaller than the wavelength, while maintaining the electromechanical coupling coefficient of the piezoelectric AlN overlayer. The propagation characteristics of very fast longitudinal modes, the Anisimkin Jr. plate modes, along wz-BN/AlN thin composite plates is studied for different BN and AlN thicknesses and electrical boundary conditions. The study of the S_0_ mode phase velocity and coupling coefficient K^2^ dispersion curves revealed that eight different coupling configurations are allowable showing a K^2^ as high as about 4% and a very high phase velocity (up to about 16,700 m/s). The gravimetric sensitivity of the BN/AlN-based configurations was theoretically predicted, specifically addressing the design of enhanced coupling, microwave frequency mass sensors based on AMs and S_0_ modes, for applications in probing the solid/liquid interface and in a gaseous environment. The acoustic performances of these structures, such as the longitudinal polarization, the high K^2^ and remarkable high phase velocity, suggests the novelty and the advantages of the AlN and BN combination, suitable for the development of a small, completely integrated sensor for monitoring the liquid properties. BN/AlN is a very promising substrate material suitable for developing GHz-band devices with enhanced electroacoustic coupling efficiency and suitable for working in a liquid environment.

## Figures and Tables

**Table 1.** Electromechanical coupling and phase velocity of the Rayleigh-like SAW, phase velocities of the longitudinal (LBAW), shear vertical (SVBAW) and shear horizontal (SHBAW) bulk acoustic waves propagating along the <100> direction of AlN and wz-BN crystals.

**Material**	**K^2^ (%)**	**SAW (m/s)**	**LBAW (m/s)**	**SHBAW (m/s)**	**SVBAW (m/s)**
c-AlN	0.28	5,607	10,287	5,814	6,089
wz-BN	0.04	9,719	16,781	11,027	10,587

**Table 2.** The maximum K^2^ value obtainable with the most efficient coupling structures, STFM and MSTFM, is listed together with the corresponding phase velocity and mass loading sensitivities, S and S′, evaluated with respect to the AlN and BN surfaces.

**Configuration**	$\overset{{\text{h}}_{\text{BN}}/\lambda }{\;}/\underset{{\text{h}}_{\text{AlN}}/\lambda }{\;}$	**K^2^_max_ (%)**	**v (m/s)**	**S (10^−4^ m^2^/Kgλ)**	**S**′ **(10^−4^ m^2^/Kgλ)**
STFM	0.1/0.34	3.24	9,754	−4.2	−0.87
	0.2/0.31	3.70	9,885	−5.0	−0.75
	0.3/0.28	3.92	10,204	−5.6	−0.66
	0.325/0.27	3.94	10,351	−5.8	−0.61

MSTFM	0.1/0.32	2.56	9,941	−4.5	−0.66
	0.2/0.3	3.55	10,024	−5.1	−0.61
	0.3/0.29	3.90	10,200	−5.6	−0.79
	0.325/0.274	3.94	10,284	−5.8	−0.67

**Table 3.** The highest AlN thicknesses allowing the AM propagation, the K^2^, the wave velocity and the gravimetric sensitivities, S and S′, of the most efficient coupling configurations, referring to the AlN and BN sensing side.

$\overset{{\text{h}}_{\text{BN}}/\lambda }{\;}/\underset{{\text{h}}_{\text{AlN}}/\lambda }{\;}$	**K^2^ (%)**	**v (m/s)**	**S (10^−4^ m^2^/Kgλ)**	**S′ (10^−4^ m^2^/Kgλ)**	**Configuration**
0.1/0.013	0.25	16,132	−12.30	−10.90	MSTFM
0.2/0.01	0.17	16,470	−6.92	−6.20	MSTF
0.3/0.004	0.14	16,664	−4.72	−4.40	MSTF
0.325/0.001	0.14	16,710	−4.20	−4.11	MSTF
